# The Effect of Replication Protein A Inhibition and Post-Translational Modification on ATR Kinase Signaling

**DOI:** 10.21203/rs.3.rs-4570504/v1

**Published:** 2024-07-26

**Authors:** Matthew R. Jordan, Greg G. Oakley, Lindsey D. Mayo, Lata Balakrishnan, John J. Turchi

**Affiliations:** Indiana University School of Medicine; University of Nebraska Medical Center; Indiana University School of Medicine; Indiana University Indianapolis; Indiana University School of Medicine

## Abstract

The ATR kinase responds to elevated levels of single-stranded DNA (ssDNA) to activate the G2/M checkpoint, regulate origin utilization, preserve fork stability, and allow DNA repair towards ensuring genome integrity. The intrinsic replication stress in cancer cells makes this pathway an attractive therapeutic target. The ssDNA that drives ATR signaling is sensed by the ssDNA-binding protein replication protein A (RPA), which acts as a platform for ATRIP recruitment and subsequent ATR activation by TopBP1. We have developed chemical RPA inhibitors (RPAi) that block RPA-ssDNA interactions, termed RPA-DBi, and RPA protein-protein interactions, termed RPA-PPIi; both activities are required for ATR activation. Here, we employ a biochemically reconstituted ATR kinase signaling pathway and demonstrate that both RPA-DBi and RPA-PPIi abrogate ATR-dependent phosphorylation of downstream target proteins. We demonstrate that RPA post-translational modifications (PTMs) impact ATR kinase activation but do not alter sensitivity to RPAi. Specifically, phosphorylation of RPA32 and TopBP1 stimulate, while RPA70 acetylation has no effect on ATR phosphorylation of target proteins. Collectively, this work reveals the RPAi mechanism of action to inhibit ATR signaling that can be regulated by RPA PTMs and offers insight into the anti-cancer activity of ATR pathway targeted cancer therapeutics.

## INTRODUCTION

Genomic material must be completely replicated in coordination with cell cycle progression to ensure faithful segregation of DNA to daughter cells; the passage of under-replicated chromosomes through mitosis leads to the loss of genetic material and disease^1^. Endogenous and exogenous DNA lesions are barriers to replication and can delay DNA replication. For example, the formation of DNA adducts by UV irradiation or cisplatin stalls replication forks and uncouples DNA polymerase and helicase activities^2^. This uncoupling induces replication stress, which is manifested by increased levels of single-stranded DNA (ssDNA) that must be protected before being replicated once replication fork progression has been restored^3^. Notably, cancer cells have intrinsically elevated levels of replication stress due to oncogene-driven cell cycle progression, altered DNA replication check points, defects in the DNA damage response (DDR), or other oncogenic features^4^. The ataxia telangiectasia and Rad3-related (ATR) kinase is one of the three major phosphatidylinositol 3-kinase (PI3K)-like kinases that coordinate the DDR that is responsible for directing the cellular response to replication stress to ensure complete replication of the genome before entering mitosis^5^. Thus, targeting this pathway is a viable therapeutic strategy for the treatment of cancer and has resulted in the clinical evaluation of a plethora of ATR inhibitors (ATRi) that target the kinase domain for competitive inhibition^6^. This is particularly notable as recent studies implicate ssDNA gap accumulation as the initiating lethal chemotherapeutic DNA lesion that drives the sensitivity of BRCA-deficient cancers to PARP inhibition rather than the double-strand break (DSB)^7–12^.

ATR is recruited to stretches of ssDNA by ATR interacting protein (ATRIP), which directly binds replication protein A (RPA)-bound ssDNA^13,14^. At stalled replication forks, DNA topoisomerase 2-binding protein 1 (TopBP1) is loaded onto ssDNA-dsDNA junctions^15,16^ and stimulates ATR kinase activity through protein-protein interactions via an ATR activating domain (AAD)^13,17,18^. More recently, Ewing’s tumor-associated antigen 1 (ETAA1), which also possesses an AAD, has been suggested to activate ATR and bind to RPA^19–21^, but appears to function in mitosis rather than S phase^22^. The most well-established ATR phosphorylation target is checkpoint kinase 1 (CHK1), which halts S/G2 progression and suppresses origin firing, thereby reducing the generation of new ssDNA^23,24^. Phosphorylation of the many other ATR targets regulates function in replication fork reversal and restart, maintenance of dNTP pools, and overall replisome regulation^5^. Collectively, the ATR signaling pathway decreases the production of ssDNA to maintain genomic stability until normal replicative function can be restored. When the ATR pathway is pharmacologically inhibited, ssDNA accumulates and is bound by RPA resulting in no free, unbound RPA. This phenomenon was termed RPA exhaustion and induces replication catastrophe that ultimately leads to cell death^25,26^. The inability to protect newly generated ssDNA, results in degradation and replication fork breakage occurs^25,26^. Importantly, the ATR signaling pathway, which prevents ssDNA accumulation and RPA exhaustion, is also dependent upon RPA binding to ssDNA and interacting with ATRIP to serve as a platform for kinase activation^13^. RPA is therefore central ATR signaling, executing cell cycle checkpoints and protecting ssDNA to preserve genomic stability.

RPA is the major mammalian ssDNA-binding protein and plays a central role in DNA replication, recombination, repair, and the DDR. RPA is a heterotrimer made up of RPA70, RPA32, and RPA14 subunits that contain 6 oligonucleotide/oligosaccharide-binding (OB) domains which dictate high affinity ssDNA binding and protein-protein interactions^27–29^. Because RPA is abundant (~ 4 million molecules per cell)^30^ and possesses a diffusion-limited rate of association for ssDNA binding^31^, nearly all generated ssDNA is quickly bound and protected from degradation by RPA. The RPA-ssDNA complex then serves as a hub for protein-protein interactions to recruit DNA replication/repair machinery to ssDNA. In response to various forms of DNA damaging agents, RPA is subjected to numerous post-translational modifications (PTMs) that, at times, dictate specific protein-protein interactions for certain repair pathways^32^. Phosphorylation of the RPA32 N-terminus has been well characterized; cyclin-dependent kinases phosphorylate Ser23 and Ser29 in a cell cycle-dependent manner^33^. Additionally, PI3K-like kinases phosphorylate RPA32 in response to DNA damage: ATR phosphorylates Ser33 and DNA-dependent protein kinase (DNA-PK) and ataxia telangiectasia mutated (ATM) have both been demonstrated to phosphorylate Ser4, Ser8, and Thr21^34,35^. These phosphorylation events impact DNA replication and repair, and function to maintain genomic stability^36^. Moreover, multiple acetyltransferases can acetylate RPA70 and acetylation, dependent on the particular sites, positively or negatively impacts ssDNA binding, nucleotide excision repair (NER), and homologous recombination^37–41^. Notably, RPA70 acetylation is increased upon UV damage; under these conditions, UV adducts stall replication forks thereby leading to ssDNA gap accumulation that can activate ATR^42,43^. Other RPA PTMs include methylation, ubiquitylation, SUMOylation, and ADP-ribosylation, all of which have been linked to the DDR^32^.

Due to its central role in DNA metabolism and the DDR, the chemical exhaustion of RPA through the use of a small molecule RPA inhibitors (RPAi) is a promising anti-cancer therapeutic avenue for single agent or combination treatment. Our groups and others have developed RPAi that function through distinct mechanisms. One class blocks RPA-ssDNA binding (RPA-DBi) and exhibits potency across a variety of cancer cell lines, and slows tumor growth in xenograft models^44–47^. Moreover, RPA-DBi demonstrate synergy with DNA damaging agents and other DDR inhibitors (PARP, DNA-PK, and ATR)^9,47^. The second class (RPA-PPIi) targets the RPA70 OB-F protein-protein interaction domain and has no effect on RPA ssDNA binding but disrupts the recruitment of DDR machinery and exhibits anti-cancer activity^48–50^. Taken together, inhibition of both the RPA-ssDNA complex by RPA-DBi or the RPA-ATRIP interaction by RPA-PPIi are expected to inhibit ATR kinase activity by targeting critical interactions. These hold the potential for greater selectivity and impact on the DDR/replication stress response than ATR inhibitors currently in the clinic. RPA inhibition is therefore anticipated to simultaneously abrogate ATR activity and decrease the cellular ssDNA protection threshold^47^.

In this work, we have employed the reconstituted ATR kinase signaling pathway *in vitro* using purified proteins^51–53^. We first establish the RPA-bound ssDNA- and TopBP1-dependence of ATR activity using p53 and RPA32 as phosphorylation targets. We then demonstrate RPA-DBi and RPA-PPIi mediated inhibition of ATR activity. Lastly, we examine the effect of two DNA damage-induced RPA PTMs, phosphorylation and acetylation, on ATR activity. We find that phosphorylated RPA and phosphorylated TopBP1 stimulate ATR activity, while acetylated RPA has no change in ATR activation relative to unmodified RPA. In addition, despite the increased ssDNA binding affinity upon RPA acetylation, both acetylated and unmodified RPA are inhibited by RPA-DBi to the same degree. Collectively, this work establishes a novel mechanism of action of two distinct classes of RPAi and provides new insights into the effects of RPA PTMs on ATR kinase activity.

## RESULTS

### Reconstitution of ATR Signaling and Inhibition by RPA-DBi

**Figure 1:**
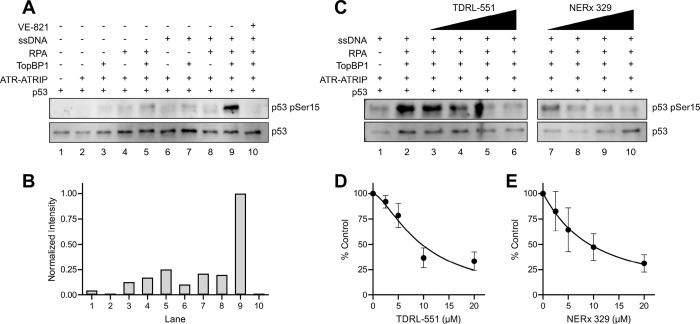
ATR phosphorylation of p53 and RPA-DBi impact. (A) Representative western blot of control reactions of the ATR kinase reconstitution demonstrating the dependence of RPA, TopBP1, and ssDNA on ATR phosphorylation of p53 Ser15. (B) Quantification of band intensities from panel A (normalized to lane 9). (C) Representative western blot of ATR-dependent phosphorylation of p53 upon increasing amounts of TDRL-551 and NERx 329 (2.5–20 μM). (D) Quantification of TDRL-551 inhibition of ATR-dependent phosphorylation events. (E) Quantification of NERx 329 inhibition of ATR-dependent phosphorylation events. Data in panels D-E are from triplicate experiments (mean ± SEM) normalized to the control reaction (panel C, lane 2) and are fit to a nonlinear regression.

The minimal requirements to reconstitute ATR signaling *in vitro* are ssDNA, RPA, TopBP1, and ATR-ATRIP, and a phosphorylation target^51–54^. We used a similar experimental design with purified RPA, TopBP1, p53, and ATR-ATRIP, and reconstituted ATR activation and signaling using an M13mp18 ssDNA plasmid (Figure S1A-B). It has been reported that TopBP1 can stimulate ATR activity independent of RPA and in the presence or absence of a variety of DNA substrates at specific protein and salt concentrations, though the physiological relevance is unknown^51^. We, therefore, first established physiological experimental conditions to reflect the dependence of ATR kinase activity on RPA, ssDNA, and TopBP1 by monitoring the phosphorylation of the ATR target p53 at Ser15. Low, background levels of ATR-dependent p53 phosphorylation are observed with various combinations of ssDNA, RPA, and TopBP1, however, as expected, maximal activation is observed only in the presence of all three (Fig. 1A–B). Importantly, p53 phosphorylation under our assay conditions was dependent upon RPA and ssDNA. All observed kinase activity was ATR-dependent as no p53 phosphorylation is observed when reactions include the ATR inhibitor VE-821^55,56^ (Fig. 1A–B). The RPA70 OB-F domain interacts with p53, and this interaction is thought to facilitate the recruitment of p53 for ATR phosphorylation^53^. p53 also has weak ssDNA binding affinity that could influence it as a substrate for ATR, so we next compared the phosphorylation of wild-type (WT) p53 with that of the DNA-binding-impaired p53 R175H mutant. We found there was no observable difference in the phosphorylation of Ser15 upon titrating WT p53 and p53 R175H (Figure S1C) demonstrating that p53 DNA binding activity is not required for ATR kinase-dependent phosphorylation of Ser15. We then evaluated the impact of chemical RPA inhibition on ATR-dependent p53 phosphorylation in the reconstituted reactions. We first assessed the activity of RPA inhibitors that block the RPA-ssDNA interaction (RPA-DBi). The TDRL-551^45^ and propyl morpholino derivative NERx 329^46^ are potent *in vitro* RPA-DNA binding inhibitors (IC_50_ ≈ 15 μM and 5 μM, respectively), and both exhibited titratable inhibition of ATR-dependent p53 phosphorylation (Fig. 1C–E). These data demonstrate that chemical RPA inhibition of its interaction with DNA disrupts ATR kinase activity.

**Figure 2:**
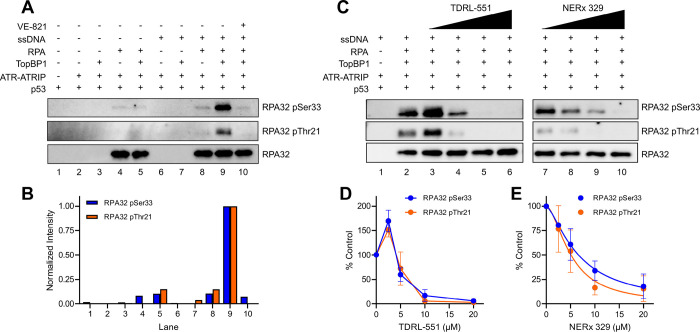
ATR phosphorylation of RPA32 and RPA-DBi impact. (A) Representative western blot of control reactions of the ATR kinase reconstitution demonstrating the dependence of RPA, TopBP1, and ssDNA on ATR phosphorylation of RPA32 Ser33 and Thr21. (B) Quantification of band intensities from panel A (normalized to lane 9). (C) Representative western blot of ATR-dependent phosphorylation of RPA upon increasing amounts of TDRL-551 and NERx 329 (2.5–20 μM). (D) Quantification of TDRL-551 inhibition of ATR-dependent phosphorylation events. (E) Quantification of NERx 329 inhibition of ATR-dependent phosphorylation events. Data in panels D-E are from triplicate experiments (mean ± SEM) normalized to a control reaction (panel C, lane 2) and data in panel E are fit to a nonlinear regression.

In addition to p53, ATR phosphorylates RPA32 on Ser33 and possibly Thr21 as part of the DDR^57,58^. Similar to the results obtained with p53 Ser15, phosphorylation of both RPA sites was dependent on ssDNA and TopBP1, with little or no observable ATR kinase activity in the absence of either (Fig. 2A–B). RPA32 Thr21 phosphorylation was weaker than that of RPA32 Ser33 likely due to non-specific ATR kinase activity towards an ATM/DNA-PK phosphorylation site (Fig. 2A). Moreover, these phosphorylation events were likewise abolished by ATR inhibitor VE-821 (Fig. 2A–B). As previously reported, increasing amounts of p53 inhibits the phosphorylation of both RPA32 sites through a noncompetitive mechanism that may be due to RPA recruitment of p53 to ATR (Figure S1C)^53^. Unexpectedly, while WT p53 and the p53 R175H DNA binding mutant were similarly phosphorylated by ATR, reactions utilizing p53 R175H did not exhibit the same inhibition of RPA32 phosphorylation as compared to WT p53 (Figure S1C). As observed with p53 phosphorylation, titration with RPA-DBi TDRL-551 and NERx 329 inhibited ATR kinase activity, resulting in dose-dependent inhibition of RPA phosphorylation (Fig. 2C–E). Interestingly, low concentrations of TDRL-551 stimulated RPA phosphorylation at both RPA32 Ser33 and Thr21, which was not observed with p53 Ser15 phosphorylation (Fig. 2C, lane 3 compare with Fig. 1C, lane 3).

The fully reconstituted ATR kinase reaction is inhibited by RPA-DBi with similar potencies towards both p53 and RPA phosphorylation. IC_50_ values for all three phosphorylation events are between 5–10 μM for both TDRL-551 and NERx 329, demonstrating that inhibition of RPA ssDNA binding directly disrupts ATR kinase activity. In addition to phospho-antibodies to p53 Ser15, RPA32 Ser33, and RPA32 Thr21, we also used phospho-antibodies to TopBP1 Ser1138 (potential DNA-PK/ATM phosphorylation site) and RPA32 Ser4/8 (DNA-PK/ATM phosphorylation sites) and no phosphorylation was detected at these sites.

### RPA-PPIi Inhibit ATR

**Figure 3:**
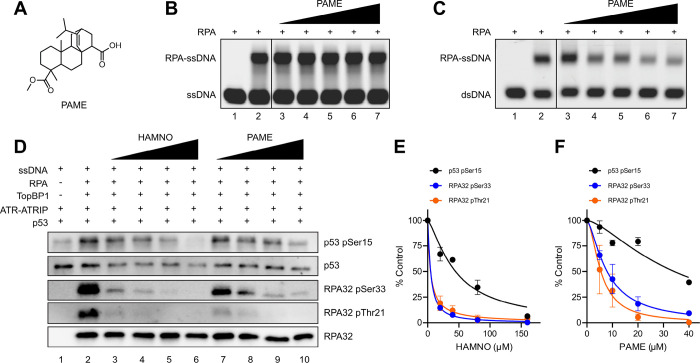
Impact of RPA-PPIi on ATR kinase activity. (A) Chemical structure of the RPA-PPIi PAME. (B) Representative EMSA of RPA ssDNA binding upon increasing amounts of PAME (12.5–200 μM). (C) Representative EMSA of RPA dsDNA unwinding upon increasing amounts of PAME (1.6–25 μM). (D) Representative western blot of ATR-dependent phosphorylation events upon increasing amounts of RPA70 OB-F inhibitors HAMNO (20–160 μM) and PAME (5–40 μM). (E) Quantification of HAMNO inhibition of ATR-dependent phosphorylation events. (F) Quantification of PAME inhibition of ATR-dependent phosphorylation events. Data in panels E-F are from duplicate experiments (mean ± range) normalized to a control reaction (panel D, lane 2) and are fit to a nonlinear regression.

With confirmation that RPA ssDNA binding inhibitors disrupt ATR signaling, we next evaluated the impact of blocking the RPA protein-protein interactions using small molecules designed towards the RPA70 OB-F domain (RPA-PPIi). This OB-fold possesses a basic cleft that is responsible for interactions with ATRIP, p53, BLM, WRN, MRE11, RAD9, and ETAA1^59^. The previously characterized small molecule HAMNO^50^ and PAME (Fig. 3A), a novel methyl ester derivative of the reported inhibitor fumaropimaric acid^49^, are predicted to bind the RPA70 OB-F basic cleft and therefore block ATRIP binding and ATR recruitment; indeed, cells treated with HAMNO have lower levels of ATR phosphorylation events^50^. Like HAMNO^50^, PAME did not disrupt RPA ssDNA binding (Fig. 3B) but did inhibit the OB-F-dependent dsDNA unwinding property of RPA (Fig. 3C), demonstrating target specificity for the RPA70 OB-F domain over the major ssDNA-binding OB-A and OB-B domains. To directly test whether RPA-PPIi disrupts the ATR signaling pathway, we titrated increasing amounts of HAMNO and PAME into the fully activated ATR reaction (Fig. 3D). Both compounds inhibited phosphorylation of p53 Ser15, RPA32 Ser33, and RPA32 Thr21 (Fig. 3D–F). Notably, phosphorylation of p53 and RPA32 were differentially affected by both RPA70 PPI inhibitors, as the IC_50_ for RPA32 phosphorylation at both residues was ~ 10-fold lower than that of p53 phosphorylation (Fig. 3E–F) and may be due to the disruption of interactions between the RPA70 OB-F and p53^49^. This is an interesting distinction between the two classes of inhibitors and their differing mechanisms of action.

### Phosphorylated RPA and TopBP1 Stimulate ATR

**Figure 4:**
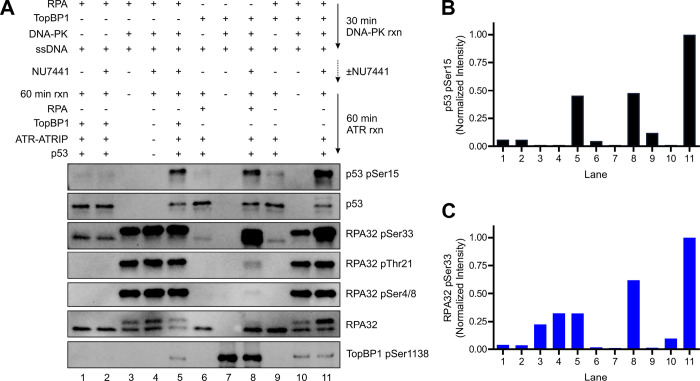
ATR activation by pRPA. (A) Representative western blot of ATR-dependent phosphorylation of p53 and RPA32 utilizing pRPA and/or pTopBP1. RPA and/or TopBP1 were initially phosphorylated by DNA-PK before being used in ATR kinase reactions as indicated by the scheme in the top right. (B) Quantification of the p53 pSer15 band intensities from panel A (normalized to lane 11). (C) Quantification of the RPA32 pSer33 band intensities from panel A (normalized to lane 11).

RPA is phosphorylated by all three PI3K-like kinases in response to different forms of DNA damage and thus these phosphorylated forms of RPA are likely the physiological platform for ATR-ATRIP recruitment^36,58,60–63^. Therefore, we evaluated how RPA phosphorylation affects ATR kinase activity, which has only been biochemically examined within the context of an RPA32 mutant containing phosphomimetic substitutions of all Ser/Thr residues within the N-terminus rather than physiological PTM at PI3K-like kinase sites^53^. Therefore, we designed an experiment where RPA32 was first phosphorylated with purified DNA-PK in a 30-minute reaction or mock-treated in the absence of DNA-PK, followed by the addition of the DNA-PK inhibitor NU7441 to halt further DNA-PK-dependent phosphorylation. Then, ATR, TopBP1, and p53 were added to evaluate ATR-dependent activation using the phosphorylated RPA (pRPA) as the DNA damage sensing platform (Fig. 4). In addition to pRPA, we also examined the effect of phosphorylated TopBP1 (pTopBP1) on ATR activation, as cellular assays have shown that phosphomimetic mutants of TopBP1 Ser1138 enhance the phosphorylation of ATR targets^64,65^. Multiple controls were included to i) establish baseline ATR phosphorylation activity using mock treated RPA and/or TopBP1 as these reactions exhibit reduced activation due to the initial 30 min incubation at 37°C in the absence of DNA-PK (Fig. 4A, lanes 1, 6, 9), ii) ensure that the concentration of NU7441 does not cause off-target inhibition of ATR-dependent phosphorylation of p53 Ser15 or RPA32 Ser33 (Fig. 4A, lanes 1 vs 2), iii) confirm that the amount of NU7441 used is sufficient to inhibit additional DNA-PK-dependent phosphorylation of RPA32 Ser4/8, Thr21, and Ser33 for the duration of the second 60 min reaction (Fig. 4A, lanes 3 vs 4), and iv) establish the level of RPA and TopBP1 phosphorylation generated by the DNA-PK reaction for pRPA and/or pTopBP1 reactions (Fig. 4A, lanes 3, 7, 10). Under these conditions, the pRPA generated by reaction with DNA-PK was representative of physiological, DDR-mediated pRPA, as we observed robust phosphorylation on RPA32 Ser33, Thr21, and Ser4/8 and 40–45% of the total RPA32 species consisted of the slower migrating, hyperphosphorylated RPA32 band (Fig. 4A, lanes 3, 10). pRPA stimulated ATR-dependent phosphorylation of p53 Ser15 by ~ 8-fold greater than that of unmodified RPA (Fig. 4, lanes 2 vs 5). Likewise, pTopBP1 increased ATR kinase activity toward p53 Ser15 ~ 8-fold, as well as RPA32 Ser33 ~ 35-fold (Fig. 4, lanes 6 vs 8). Lastly, the combination of both pRPA and pTopBP1 further stimulated ATR activity, with the highest levels of p53 pSer15 and RPA32 pSer33 observed (Fig. 4, lane 11). Notably, the pRPA reaction conditions do not yield 100% conversion to pRPA, as made apparent by the slower migrating, hyperphosphorylated band that accounts for ~ 40–45% of the total RPA32 (Fig. 4A, RPA32 panel, lanes 3–5). Additionally, in the combination reactions (Fig. 4A, lanes 9–11), TopBP1 and RPA compete as DNA-PK substrates and thus both initially have lower levels of phosphorylation relative to reactions where they are the sole phosphorylation target, particularly TopBP1 pSer1138 (Fig. 4A, lanes 3–5 vs 10–11 for pRPA comparison, lanes 7–8 vs 10–11 for pTopBP1 comparison). Therefore, our results likely represent an underestimation of the activation by pRPA and/or pTopBP1, and reaction conditions yielding fully phosphorylated RPA and TopBP1 would exhibit further stimulation.

### Acetylated RPA Supports ATR Function

**Figure 5:**
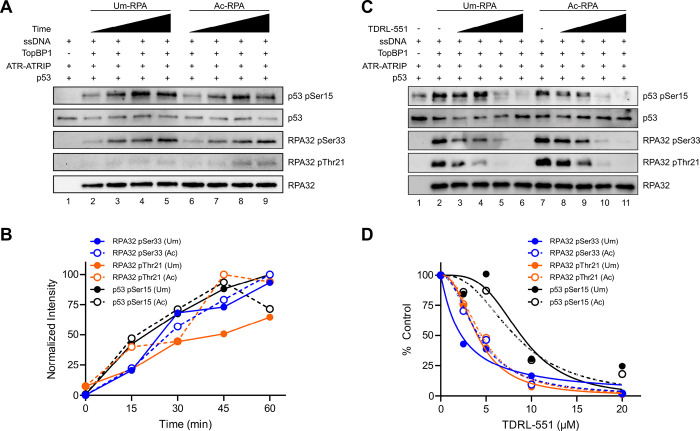
ATR activation by AcRPA and RPA-DBi impact. (A) Representative western blot of ATR-dependent phosphorylation events over time utilizing Um- or Ac-RPA. (B) Quantification of band intensities from panel A (normalized to lanes 5 and 9 for Um- and Ac-RPA, respectively). (C) Representative western blot of ATR-dependent phosphorylation events upon increasing amounts of RPA-DBi TDRL-551 (2.5–20 μM) utilizing Um- or Ac-RPA. (D) Quantification of band intensities from panel C (normalized to lanes 2 and 7 for Um- and Ac-RPA, respectively).

RPA70 is acetylated in S phase, where the ATR kinase pathway is active, in response to UV irradiation^39–41^, which generates UV adducts and large ssDNA gaps that serve as RPA-bound ATR substrates. While NuA4-induced acetylation of yeast RPA70 and human RPA70KQ mutants showed decreased binding to ssDNA^37,38^, our recent studies have shown that p300 activity results in RPA lysine acetylation at six sites and increases ssDNA binding affinity (Figure S2A-C)^41^, which could impact ATR-ATRIP recruitment and potentially sensitivity to chemical RPA inhibition. Therefore, we evaluated the impact of RPA acetylation on ATR signaling by performing an *in vitro* acetylation reaction with RPA, acetyl-CoA, and p300 (Ac-RPA) and comparing ATR-dependent phosphorylation reactions containing Ac-RPA with reactions containing unmodified RPA (Um-RPA, mock treated with no acetyl-CoA and p300)^41^. Acetylation of RPA resulted in no difference in ATR kinase activation (Fig. 5A–B, S2D-F), similar to results observed in yeast cells upon DSB induction^38^. Three p300 acetylation sites are located near or within the RPA70 OB-A and OB-B that are primarily responsible for high affinity ssDNA binding and are the predicted RPA-DBi binding domain (Figure S2A), therefore we assessed RPA-DBi sensitivity between Um-RPA and Ac-RPA. ATR kinase activity dependent on both forms of RPA was similarly inhibited by RPA-DBi (Fig. 5C–D, S2G-I), demonstrating that RPA-DBi effectiveness is unchanged towards Ac-RPA found in S phase and in response to DNA damaging agents^41^. Interestingly, we occasionally did observe an increase in ATR activation by Ac-RPA (Figure S2J). Nonetheless, the degree of RPA-DBi inhibition was unchanged in these cases (Figure S2K-L). These data suggest that RPA inhibition is effective independent of RPA acetylation and explain the cellular activity of RPA-DBi in combination with DNA damaging agents where synergy has been observed *in vitro* and *in vivo*^45^.

## DISCUSSION

The work described here has characterized a novel mechanism of action for ATR inhibition by targeting the ssDNA sensor and recruitment platform RPA. RPAi offer a selectivity advantage over ATP-competitive ATRi with potential off-target effects within the kinome^66,67^. Chemical RPA exhaustion, as opposed to ATRi-induced RPA exhaustion, has the further effect of disrupting ATR kinase activity and reducing the RPA protection threshold of ssDNA from replication catastrophe. Moreover, RPA is intimately connected to nearly every facet of DNA metabolism and is therefore a wider-spanning, advantageous target for the treatment of genomically unstable cancer cells.

DSBs have long been thought to be the toxic DNA lesion conferring sensitivity to PARPi in BRCA-deficient cancers. However, recent investigations into the synthetic lethality of PARP inhibition (PARPi) in BRCA-deficient cells suggest replication-dependent lagging strand ssDNA gaps significantly contribute to the therapeutic response^7,9,10^. These ssDNA gaps must be bound by RPA to protect from nuclease degradation and enable gap filling via DNA Pol θ or re-initiation via Primpol to preserve genomic integrity and enable completion of S-phase replication. RPA-bound gaps then serve as the hub for ATR recruitment and subsequent activation by TopBP1 to signal downstream effectors that function to execute cell cycle checkpoints, prevent additional ssDNA from being generated, and allow effective creation of dsDNA at the gap to reduce the cellular ssDNA levels. Pharmacological inhibition of ATR causes excessive amounts of ssDNA such that the cellular RPA is ssDNA-bound and exhausted and can induce replication catastrophe and ultimately cell death^25,26^. Moreover, ATR inhibition is synergistic with ssDNA-gap-inducing PARPi in a process proposed to be due to exacerbated gap accumulation that exhausts all free RPA^9^. Thus, this feedback loop of RPA protection of ssDNA and subsequent ATR signaling to limit ssDNA generation relative to the RPA protection threshold suggests that the inhibition of RPA ssDNA binding and protection will further exacerbate the toxic effects of ssDNA gaps. Indeed, RPA-DBi are synthetic lethal in BRCA-deficient cells and synergize with PARPi^9,47^. RPA-PPIi have been shown to inhibit ATR-dependent phosphorylation events in cells^50^, and have been confirmed to directly inhibit ATR kinase activity in this work. Therefore, mechanistically, RPA-PPIi enhances replication stress through the disruption of ATR-dependent alleviating pathways. Alternatively, RPA-DBi have the double effect of ATR inhibition while also leaving ssDNA unprotected via chemical RPA exhaustion.

The RPA32 N-terminus is phosphorylated during S phase and in response to various forms of DNA damage, and functions in ssDNA binding, protein-protein interactions, DNA synthesis, and the replication stress response^36,58,60,68^. The relationship between phosphorylation sites is complex; phosphorylation of some residues primes other residues for phosphorylation^69–71^. Focusing on ATR kinase activity and the RPA32 Ser33 ATR site specifically, mutation to Ala results in reduced phosphorylation of Ser29 (CDK) and Ser4/8 (DNA-PK/ATM), but not Thr21 (DNA-PK), suggesting that ATR phosphorylation events prime other PI3K-like kinase sites^69^. Conversely, ATR activity is altered by other phosphorylation events as Ser33 phosphorylation is decreased upon mutation of Ser23 (CDK) or Thr21^69^. As Ser33 is typically phosphorylated before Ser4/8 in response to some DNA damaging agents, it has been difficult to ascertain the priming effects that RPA phosphorylation has on ATR activity or the ATR-specific site^71^. Recent work also implicates Ser4/8 phosphorylation in priming ATR activity; however this effect was observed for RPA32 Ser33 and not other ATR targets (CHK1) and is further complicated by differences in phosphorylation dependent upon the cell cycle phase^70,71^. Nonetheless, our results here demonstrate that phosphorylated RPA stimulates ATR kinase activity, although the specific contribution towards RPA32 Ser33 could not be definitively established due to phosphorylation of by DNA-PK at multiple sites. This notably differs from other studies where an RPA32 mutant containing eight phosphomimetic Asp substitutions (pmRPA) in the RPA32 N-terminus exhibited reduced levels of ATR activation relative to WT RPA32^53^. The basic cleft of the RPA70 OB-F domain that is responsible for interactions with numerous DDR proteins also interacts with the RPA32 N-terminus when phosphomimetic mutants are introduced, with increasing binding affinity with increasing Asp insertion^63,72^. Therefore, it is possible that the insertion of pmRPA enables a stable complex with the RPA70 OB-F domain that completely excludes ATRIP binding and subsequent ATR activation, akin to the inhibition of DNA resection by pmRPA likely due to abolished OB-F-dependent interactions of pmRPA with BLM^73^. Alternatively, the four RPA32 N-terminus phosphorylation events observed under our reaction conditions are not sufficient to form a stable complex with the RPA70 OB-F domain and have a stimulatory effect through a different mechanism. Interestingly, these observations suggest biphasic activation and inhibition, where initial phosphorylation events may stimulate ATR activation, while cumulative phosphorylation is inhibitory.

The role of RPA acetylation is particularly interesting within the context of lagging strand processing. Flap endonuclease 1 (FEN1), DNA nuclease/helicase 2 (DNA2), and Pol δ have altered activities when acetylated by p300, which favor the long flap pathway over the short flap pathway in Okazaki fragment maturation^74–76^. Consistently, p300-dependent acetylation of RPA enhances ssDNA binding affinity towards shorter ssDNA substrates and may well further favor long flap processing^41^. Defects in Okazaki fragment maturation due to mutations in the nucleases (FEN1 and DNA2) can lead to greater lengthening of long flaps such that they can accommodate the binding of multiple RPA molecules and serve as a platform for ATR signaling^77,78^. Moreover, the enhanced ssDNA binding affinity of acetylated RPA, which occurs due to a decrease in off rate of ssDNA binding^41^, is expected to provide better protection of the ssDNA gaps that occur under conditions of replication stress. Importantly, while this did not lead to any difference in ATR activation by Ac-RPA consistent with the ATR response to DSBs observed in yeast cells^38^, we found that RPA-DBi are equally as potent against the modified form of RPA.

In this study, we have described a novel mechanism of chemical RPA inhibition utilizing two classes of RPA inhibitors. Inhibition of RPA ssDNA binding or OB-F-dependent protein-protein interactions prevent ATR kinase activation by negating the sensor of the ssDNA lesion. RPA inhibitors are thus indirectly ATR inhibitors. Moreover, RPA inhibitors chemically exhaust RPA in oncogenic cells with high levels of replication stress, while also inhibiting compensatory pathways that reduce the cellular ssDNA burden. For example, cells with ssDNA gap accumulation caused by defects and/or inhibition of the DDR are sensitized by sublethal doses of RPA inhibitors^9^. Taken together, the use of RPA inhibitors as RPA- and ATR-inhibiting, anti-cancer agents is multifaceted and will have significant clinical ramifications as RPA serves numerous functions in DNA metabolism.

## MATERIALS AND METHODS

### Reagents

TDRL-551 and NERx 329 were synthesized as previously described^45,46^. HAMNO ((1Z)-1-[(2-hydroxyanilino)methylidene]naphthalen-2-one) and PAME (5-methoxycarbonyl-5,9-dimethyl-16-propan-2-yltetracyclo[10.2.2.01,10.04,9]hexadic-15-ene-14-carboxylic acid) were acquired from the Developmental Therapeutics Program of the National Cancer Institute^50^. The M13mp18 single-stranded DNA plasmid used as the ssDNA substrate was purchased from Fisher Scientific (N4040S). Active ATR-ATRIP was purchased from Eurofins DiscoverX (14–953). Recombinant full-length active p300 was purchased from Active Motif (81158).

### Protein Purification

His-tagged RPA, referred to as RPA, was purified as follows. The p11d-tRPA70-His plasmid (kindly provided by Edwin Antony) was transformed into *E. coli* BL21(DE3) competent cells and plated on Luria broth (LB) agar plates supplemented with 0.1 mg/mL ampicillin overnight at 37°C. A single colony was inoculated into 5 mL LB supplemented with 0.1 mg/mL ampicillin and incubated overnight at 37°C with agitation. The saturated culture was inoculated into 1 L LB supplemented with 0.1 mg/mL ampicillin at 37°C and expression was induced with 0.3 mM (isopropyl β-D-1-thiogalactopyranoside (IPTG) once the OD_600_ = 0.5–0.8. Cells continued to grow at 37°C for 3–4 hours before they were collected by centrifugation at 4000 × g, 20 min, 4°C and stored at −80°C overnight. Cell pellets were resuspended in HI Buffer (30 mM HEPES pH 7.8, 10% glycerol, 0.02% Tween20, 1 mM phenylmethylsulfonyl fluoride [PMSF], 5 μg/mL leupeptin, 2 μg/mL pepstatin A, 1 mM 2-mercaptoethanol [BME]) containing 50 mM KCl and 0.25 mM EDTA. Lysozyme was added to 1 mg/mL and incubated on ice for 30 min. The cell suspension was then sonicated for 10 min total (cycles of 15 sec on, 45 sec off). The suspension was centrifuged at 10000 × g, 20 min, 4°C and the soluble cell extract was loaded onto a Blue column pre-equilibrated with HI Buffer containing 50 mM KCl. The column was washed sequentially with HI Buffer containing 50 mM KCl, 0.8 M KCl, 0.5 M NaSCN, and 1.5 M NaSCN. The last, RPA-containing 1.5 M NaSCN wash was then loaded onto a hydroxyapatite column pre-equilibrated with HI Buffer. After washing with HI Buffer, RPA was eluted with HI Buffer containing 80 mM potassium phosphate. The elution was loaded onto a NiNTA column pre-equilibrated with HI Buffer containing 300 mM KCl and 10 mM imidazole. The column was washed with HI Buffer containing 300 mM KCl and 10 mM imidazole and RPA was eluted with HI Buffer containing 300 mM KCl and 400 mM imidazole. The elution was dialyzed into HI buffer containing 100 mM KCl, aliquoted, and stored at −80°C.

GST- and His-tagged TopBP1, referred to as TopBP1, was purified as follows^79^. The plasmid GST TopBP1 (aa 32–1522) His was a gift from Aziz Sancar (Addgene plasmid # 20375 ; http://n2t.net/addgene:20375 ; RRID:Addgene_20375) and was transformed into *E. coli* BL21(DE3) competent cells and plated on LB agar plates supplemented with 0.1 mg/mL ampicillin overnight at 37°C. A single colony was inoculated into 5 mL LB supplemented with 0.1 mg/mL ampicillin, and incubated overnight at 37°C with agitation. The saturated culture was inoculated into 1 L LB supplemented with 0.1 mg/mL ampicillin at 37°C and expression was induced with 0.1 mM IPTG once the OD_600_ = 0.5–0.8. The temperature was lowered to 25°C and the cells continued to grow for 5 hours before they were collected by centrifugation at 4000 × *g*, 20 min, 4°C and stored at −80°C overnight. Cell pellets were resuspended in 50 mM Tris pH 7.5, 1 M NaCl, 10 mM imidazole, 0.1% Triton X-100, 10 mM BME, 1 mg/mL lysozyme, 1 mM PMSF, 5 μg/mL leupeptin, 2 μg/mL pepstatin A and lysed by sonication for 10 min total (cycles of 15 sec on, 45 sec off). The suspension was centrifuged at 10000 × *g*, 20 min, 4°C and the soluble cell extract was mixed with an equivolume of PBS. The suspension was loaded onto a NiNTA column pre-equilibrated with TopBP1 Ni buffer (11.8 mM phosphate pH 8.0, 150 mM NaCl, 0.05% Triton X-100, 1 mM BME) containing 10 mM imidazole. The column was washed with TopBP1 Ni buffer and TopBP1 was eluted with TopBP1 Ni buffer containing 400 mM imidazole. Pre-equilibrated GST Sepharose slurry was added to the elution and incubated at 4°C with rocking overnight. The mixture was assembled into a column, washed with TopBP1 GST buffer (50 mM Tris pH 8.0, 100 mM NaCl, 1 mM EDTA, 10% glycerol, 0.02% Triton X-100, 0.5 mM DTT), and TopBP1 was eluted with TopBP1 GST buffer containing 10 mM reduced glutathione. The elution was dialyzed into TopBP1 GST buffer, aliquoted, and stored at −80°C.

Wild-type and mutant R175H His-tagged p53, referred to as p53, was purified as follows. The pET28a-His-p53 (and mutants) was transformed into *E. coli* Rosetta and selected for kanamycin resistance (50 μg/ml) on LB plates. Colonies were picked and grown overnight in 50 mL of LB with kanamycin in a bacterial shaker at 37°C at 200 rpm. The next day, 500 ml of LB with kanamycin was inoculated with the overnight culture until the OD_600_ = 0.5–0.8. The culture was transferred to a shaker at 25°C, and 1 mM IPTG was added and incubated at 200 rpm for 4 hours. Cells were collected by centrifugation, and the pellet was stored at −80°C freezer. The pellet was resuspended in 50 mM NaH_2_PO_4_, 250 mM KCl, 0.05% IGAPAL, and 10 mM imidazole, pH 8.0. The pellet was sonicated, and then debris was pelleted. The sample was loaded onto a NiNTA column that was pre-equilibrated with lysis buffer. The column was washed with lysis buffer in the absence of IGAPAL and the addition of 1M KCL, then washed with 10 column volumes of lysis buffer without IGAPAL. p53 was eluted in PBS with 500 mM Imidazole, dialyzed into PBS pH 7.2 with 25% glycerol, aliquoted, and stored at −80°C.

### ATR Kinase Assay

Kinase assays were performed in 15 mM HEPES pH 7.9, 30 mM KCl, 3 mM MgCl_2_, 5% glycerol, 1x phosphatase inhibitor cocktail 2 (Sigma-Aldrich, P5726), 0.5 mM DTT. RPAi were suspended in 100% DMSO, and the DMSO concentration in the final reaction mixture was kept constant at 5%. In a typical reaction (20 μL), 20 ng ATR-ATRIP, 20 nM TopBP1, 20 nM RPA, and 40 nM p53 were initially incubated on ice for 15 min. Reactions were initiated by the addition of 100 μM ATP and 1 ng M13mp18 ssDNA plasmid and incubated at 37°C for 1 hr. Reactions were quenched by the addition of 6x Laemmli buffer and brief boiling at 95°C. For reactions with RPAi, inhibitors were added for the initial 15 min incubation on ice. For reactions with pRPA, the indicated proteins (same concentrations as above) were incubated with 0.5 ng/μL DNA-PK, 100 μM ATP, and 1 ng M13mp18 ssDNA plasmid for 30 min at 37°C. Reactions were moved back to ice and 100 nM NU7441 was added to inhibit further DNA-PK kinase activity (for subsequent ATR kinase reactions) or quenched with 6x Laemmli buffer and brief boiling at 95°C (for control reactions). The remaining components required for ATR kinase reaction were then added to each reaction as indicated and returned to 37°C for 1 hr before quenching in the same manner as above. Proteins were separated by SDS-PAGE and transferred to polyvinylidene difluoride (PVDF) membranes (Bio-Rad, 1620177). Immunoblotting for RPA32 pSer33 (Thermo Fisher Scientific, A300–246A), RPA32 pSer4/8 (Thermo Fisher Scientific, A300–245A), RPA32 (Thermo Fisher Scientific, PA5–2256), and TopBP1 pSer1138 (Thermo Fisher Scientific, PA5–114666) were conducted in Tris-buffered saline (TBS)/0.5% Tween20 and immunoblotting for p53 pSer15 (Cell Signaling Technology, 9284S), p53 (Cell Signaling Technology, 2524S), and RPA32 pThr21 (Thermo Fisher Scientific, PA5–104809) were conducted in TBS/0.1% Tween20. All membranes were blocked with 2% BSA in TBS/Tween20 and incubated rocking at 4°C overnight. Membranes were washed with TBS/Tween20 and incubated with the corresponding IgG-horseradish peroxidase (HRP) secondary antibody (Bio-Rad, 1706515 & 1706516) for two hours at room temperature with orbital shaking. Membranes were washed with TBS/Tween20 and immunoblots were detected using the chemiluminescent Clarity Western ECL Substrate (Bio-Rad, 1705061).

### Electrophoretic Mobility Shift Assay (EMSA)

The EMSAs to evaluate PAME inhibition were performed as follows^50^. These EMSA reactions were conducted in 10 mM Tris pH 7.5, 10 mM NaCl, 10% glycerol. For ssDNA binding experiments, a 30 base polyT substrate containing an IRDye700 label on the 5’ end (Integrated DNA Technologies) was used. 7 nM purified RPA was added to a mixture of 10 nM labeled ssDNA and various concentrations of PAME for 10 min at 25°C. Products were separated on 1% agarose gels and visualized on an infrared scanner. For dsDNA unwinding studies, a 30mer duplex oligonucleotide was created by annealing the IRDye700-labeled polyT 30mer to a polyA 30mer (Integrated DNA Technologies). Duplex DNA was PAGE purified and eluted overnight in EMSA buffer. 14 nM purified RPA was added to a mixture of 10 nM labeled ssDNA and various concentrations of PAME for 10 min at 25°C. Products were separated on 1% agarose gels and visualized on an infrared scanner.

The EMSAs to evaluate the effect of RPA acetylation were performed as follows^46^. These EMSA reactions (20 μL) were conducted in 20 mM HEPES pH 7.8, 50 mM NaCl, 1 mM DTT, 5% DMSO, 0.001% NP-40. For the preparation of radioactively labelled ssDNA substrate (50 μL reaction), 0.2 μM of the 34 base ssDNA substrate (Integrated DNA Technologies) was incubated with 0.4 μM γ-[^32^P]-ATP and T4 PNK in T4PNK buffer at 37°C for 30 min. 20 μM ATP was added for an additional 5 min at 37°C before the reaction was quenched with the addition of 40 mM EDTA and subsequent 70°C incubation for 10 min. The [^32^P]-labeled DNA substrate was purified with a G-50 column and total yield quantified with a scintillation counter. 2.5 nM [^32^P]-labeled DNA was incubated with increasing concentrations of purified RPA for 5 min at room temperature and products were separated via 6% native polyacrylamide gel electrophoresis. The bound and unbound fractions were quantified by phosphor-imager analysis.

### Identification of PAME from High Throughput Screen

The high throughput screen (HTS) is based on RPA DNA unwinding property. Initially, a 30mer duplex oligonucleotide created by annealing a polyT 30mer containing an IRDye700 label on the 5′ end (Integrated DNA Technologies) to a polyA 30mer containing a Black Hole Quencher 3 (BHQ3) label on the 3′ end (Operon) was PAGE purified and eluted overnight in EMSA buffer (10 mM Tris pH 7.5, 10 mM NaCl, 10% glycerol). For the assay, 20 μL of 10 nM duplex DNA in EMSA buffer was first added to a clear 384-well plate. Next, 0.5 μL of 10 mM compound from the Diversity set III and Diversity set IV was added to each well and mixed. Finally, 20 μL of 10 nM RPA in EMSA buffer was added to each well and incubated for 10 min at room temperature (RT) before being scanned on an infrared scanner (Li-COR). The effective concentration of compound in this assay was 250 μM. Z-factors for the HTS were determined through an online Z-factor calculator (http://www.screeningunit-fmp.net/tools/z-prime.php [screeningunit-fmp.net]). In addition to Diversity sets III and IV, additional compounds obtained from the Developmental Therapeutics Program of the National Cancer Institute were used in identifying PAME as a compound of interest.

### In vitro RPA Acetylation

RPA acetylation was performed as previously described^41^. Briefly, reactions were performed in 50 mM Tris-HCl (pH 8.0), 150 mM NaCl, 10% glycerol, 1 mM DTT, 1 mM PMSF, 10 mM sodium butyrate. RPA was incubated with p300 and freshly prepared acetyl CoA in a 1:1:10 ratio (RPA:p300:acetyl CoA) for 30 mins at 37°C. The unmodified RPA (Um-RPA) control was treated similar to the acetylated RPA (Ac-RPA), but without the addition of acetyl-CoA. Sites of in vitro acetylated RPA70 were confirmed by LC-MS/MS mass spectrometry.

## Data Availability

All data underlying this article are available within the article and in its online supplementary material. Further inquiries can be directed to the corresponding author.
